# Pathogenic roles and therapeutic potential of the CCL8–CCR8 axis in a murine model of IgG4-related sialadenitis

**DOI:** 10.1186/s13075-021-02597-6

**Published:** 2021-08-14

**Authors:** Fumika Honda, Hiroto Tsuboi, Yuko Ono, Saori Abe, Hiroyuki Takahashi, Kiyoaki Ito, Kazunori Yamada, Mitsuhiro Kawano, Yuya Kondo, Kenichi Asano, Masato Tanaka, Marie Malissen, Bernard Malissen, Isao Matsumoto, Takayuki Sumida

**Affiliations:** 1 Departments of Internal Medicine, Faculty of Medicine, University of Tsukuba, 1-1-1 Tennodai, Tsukuba-city, Ibaraki 305-8575 Japan; 2 Department of Rheumatology, Graduate School of Medical Science, Kanazawa University, 13-1 Takara-machi, Kanazawa-city, Ishikawa 920-8640 Japan; 3 Laboratory of Immune Regulation, School of Life Science, Tokyo University of Pharmacy and Life Science, 1432-1 Horinouchi, Hachioji, Tokyo, 192-0392 Japan; 4 Centre d’Immunologie de Marseille-Luminy, Aix Marseille Université, INSERM, CNRS, 13288 Marseille, France

**Keywords:** IgG4-related disease, Animal model, Chemokine, CCL18, CCL8, Fibrosis

## Abstract

**Background:**

Our previous studies reveal that CCL18-CCR8 chemokine axis is upregulated in patients of immunoglobulin G4-related disease (IgG4-RD), suggesting that the CCL18–CCR8 axis is implicated in the etiology of IgG4-RD, although whether this axis has a potential as a therapeutic target remains unclear. Our purpose was to clarify the pathogenic roles and therapeutic potential of the murine CCL8 (analog of human CCL18)–CCR8 axis by using an animal model of IgG4-RD (LAT Y136F knockin mice; LAT mice).

**Methods:**

We compared the infiltration of inflammatory cells and the fibrosis of the salivary glands of 6-week-old LAT mice and littermate mice. The expressions of *Ccl8* and *Ccr8* were also compared. Next, we investigated the therapeutic effects of intravenous administration of anti-CCL8 neutralizing antibody in LAT mice against inflammation and fibrosis of the salivary glands. We also investigated the effects of stimulation with recombinant mouse CCL8 on the collagen production in a mouse fibroblast cell line (NIH/3 T3) in vitro*.*

**Results:**

When compared with the littermates, the LAT mice showed apparent infiltration of inflammatory cells and fibrosis in the salivary glands. The focus and fibrosis score in the salivary glands were significantly higher in the LAT mice than in the littermates. The expression levels of *Ccl8* in the spleen and of *Ccr8* in the salivary glands were significantly higher in the LAT mice than in the littermates. Anti-CCL8 antibody significantly improved the focus and fibrosis score in the salivary glands of the LAT mice. In vitro, stimulation with recombinant mouse CCL8 significantly increased the expression of collagen and ERK1/2 phosphorylation in NIH/3 T3.

**Conclusion:**

We clarified the overexpression and therapeutic potential of the mouse CCL8–CCR8 axis in LAT mice, which could play a crucial role in fibrosis via ERK1/2 phosphorylation, as well as the chemotaxis of inflammatory cells. The human CCL18–CCR8 axis might be a novel therapeutic target for IgG4-RD.

**Supplementary Information:**

The online version contains supplementary material available at 10.1186/s13075-021-02597-6.

## Background

Immunoglobulin G4-related disease (IgG4-RD) is characterized by elevation of serum IgG4 levels and infiltration of IgG4-positive plasmacytes and fibrosis of multiple organs [1, 2]. Especially in IgG4-RD sialadenitis, the cytokine environment of the salivary glands (SGs) is predominantly composed of type-2 T helper (Th2) cytokines and IL-10 expression is elevated when compared with the cytokine environment of Sjögren’s syndrome, which also presents with sialadenitis [3]. In clinical practice, glucocorticoid treatment is effective for inducing clinical remission, but relapse occurs frequently with tapering of the steroid dose [4]. Therefore, a disease-specific treatment strategy based on the etiology is required.

In our previous studies, we revealed that the chemokine (C-C motif) ligand 18 (CCL18) and its receptor chemokine (C-C motif) receptor 8 (CCR8) in labial salivary glands (LSGs) of IgG4-RD patients are upregulated when compared with those of healthy controls and of Sjögren’s syndrome patients [5]. Both IgG4-RD and Sjögren’s syndrome can cause sialadenitis and are important for differential diagnosis [6], while the upregulation of CCL18-CCR8 axis is considered to be specific to IgG4-RD. CCL18 (hCCL18) is produced mainly by macrophages and dendritic cells. It has a chemotactic function for T cells and B cells and induces the fibrosis [7, 8]. We also reported that in the LSGs of IgG4-RD patients, some macrophages and dendritic cells expressed hCCL18 [9]. In addition, the number of hCCL18-positive macrophages and dendritic cells in the LSGs of IgG4-RD patients was higher than in those of healthy controls and Sjögren’s syndrome patients [9]. Furthermore, hCCL18 specifically enhanced IgG4 production by stimulating peripheral blood mononuclear cells in vitro [9]. It was reported that serum hCCL18 concentration was correlated with disease activity and the number of involved organs [10]. These findings suggest that the hCCL18–CCR8 axis is implicated in the etiology of IgG4-RD, although whether this axis has a potential as a therapeutic target remains unclear.

Animal models of disease are essential for the development of therapeutic agents, especially in rare diseases of unknown etiology. However, rodents do not possess the IgG4 subtype, making it difficult to replicate IgG4-RD in a mouse model. The linker for activation of T cells (LAT) Y136F knockin mouse (LAT mouse) has been reported as a mouse with elevated type-2 T helper (Th2) cytokine and serum IgG1 (human IgG4-equivalent) levels [11]. In recent years, some reports showed that the LAT mouse could be an animal model of IgG4-RD [12, 13]. LAT mice showed IgG1-positive plasmacyte infiltration and fibrosis in some organs, including the SGs, as well as elevated Th2 cytokine and serum IgG1 [12]. These characteristics are held in common by human IgG4-RD patients. Furthermore, the tissue lesions of LAT mice were reduced by therapeutic administration of corticosteroid, also in common with human IgG4-RD [12]. Interestingly, it has been reported that CCR8, which is a receptor for hCCL18 and murine CCL8 (mCCL8; analog of hCCL18), could be upregulated in T cells in a Th2-polarized environment [14] and that IL-4 and IL-10 could induce the production of hCCL18 and mCCL8 from macrophages [14].

In this study, we analyzed the expression levels of the mCCL8 (analog of hCCL18)–CCR8 axis in LAT mice as a murine model of IgG4-RD and injected the mice with anti-mCCL8 antibody to explore whether mCCL8-blocking therapy would reduce the development of tissue lesions, especially in sialadenitis, in which the elevated expression of hCCL18 has already been demonstrated in human IgG4-RD.

## Materials and methods

### Animals

LAT Y136F knockin mice with a C57BL/6j background were kindly provided by Dr. Malissen (Marseille Université, Marseille, France) and maintained under specific pathogen-free conditions. All the animal protocols were approved by the Institutional Animal Experiment Committee of the University of Tsukuba (Permit No.20-189), and all the experiments were performed in accordance with relevant guidelines and regulations.

### Cell culture and treatment

The NIH/3 T3 mouse fibroblast cell line was purchased from the Cell Engineering Division of the RIKEN BioResource Center (Tsukuba, Ibaraki, Japan). The cells were cultured in Dulbecco’s modified Eagle’s medium (DMEM) containing antibiotics and 10% fetal bovine serum (FBS) at 37 °C in a humidified incubator containing 5% CO_2_. For all the experiments, the cells were used within 3 to 7 passages after the reception. For in vitro functional analysis of CCL8, NIH/3 T3 cells were seeded in a 96-well plate at a density of 1 × 10^5^ cells/mL. Some groups of cells were cultured with varying concentrations of recombinant mouse CCL8 (BioLegend, San Diego, CA) for 24 h. The concentration of FBS was reduced to 1% at 24 h before testing. Total collagen was detected by the Sirius Red Collagen Detection Kit (Chondrex, Woodinville, WA) according to the manufacturer’s instructions.

### Histopathologic analysis

The SGs were surgically excised, fixed in neutralized 10% formalin, and embedded in paraffin, and 3-μm-thick slices were prepared. The slices were stained with hematoxylin and eosin (H&E) and with Masson’s trichrome (M&T) according to the standard techniques. The inflammatory lesions were graded histologically using the focus score method proposed by Greenspan et al. [15] as follows: the focus score was described as the number of a focus per 4 mm^2^ of each section. One focus was defined as > 50 mononuclear cells found around the SG ducts. The fibrotic lesions were graded as occupancy of fibrosis per 4 mm^2^ of each section: 0, without fibrosis; 1, 1 to 24%; 2, 25 to 49%; and 3, higher than 50%. The histopathologic evaluation was performed by means of light microscopy in a blinded manner.

### Immunofluorescence and immunohistochemical staining

For confocal microscopy, the organs were embedded in optimum cutting temperature (OCT) compound (Tissue-Tek; Sakura Finetek, Tokyo, Japan) and frozen at − 80 °C. A cryostat was used to generate 5-μm slices, which were kept at − 20 °C. On the day of staining, the slices were thawed and dried and then fixed with cold acetone for 10 min. The slices were rehydrated in phosphate-buffered saline (PBS), blocked in PBS containing 1% bovine serum albumin (BSA; FujiFilm Wako Pure Chemical, Tokyo, Japan) for 30 min at 20 °C–22 °C, and stained with Alexa Fluor 488-conjugated rat anti-mouse CD4 antibody (Thermo Fisher Scientific, Waltham, MA) or with Alexa Fluor 647-conjugated rat anti-mouse B220, Alexa Fluor 488-conjugated rat anti-mouse F4/80 antibody, and Alexa Fluor 488-conjugated rat anti-mouse CD11c antibody (all from BioLegend). For double staining of CCL8 and cell surface molecules, the slices were blocked in 3% H_2_O_2_ in methanol to block endogenous biotin and stained with biotinylated anti-mouse MCP-2/CCL8 antibody (R&D Systems, Minneapolis, NE). The primary antibody was left to incubate overnight at 4 °C. Streptavidin-Alexa Fluor 594 (BioLegend) was used as a secondary antibody and was left to incubate for 30 min at 20 °C–22 °C. For staining of CCR8, the cells were applied to glass slides by use of Smear Gel (GenoStaff, Tokyo, Japan) according to the manufacturer’s instructions. After being blocked in PBS containing 1% BSA, the slices were stained with anti-mouse CCR8 antibody (Abcam, Cambridge, UK) and incubated overnight at 4 °C. Alexa Fluor 546 goat anti-rabbit IgG (H + L) (Thermo Fisher Scientific) was used as a secondary antibody. The control slides were incubated with a dilution buffer containing isotype-matched antibodies instead of primary antibodies. All the dilutions were made in Solution B (Toyobo, Osaka, Japan). The nuclei were counterstained with 4′,6-diamidino-2-phenylindole (DAPI). All the slides were mounted in Fluorescence Mounting Medium (Dako Carpinteria, Santa Barbara, CA). For immunohistochemical staining, 5-μm slices were cut from formalin-fixed and paraffin-embedded tissues, deparaffinized, rehydrated, blocked in PBS containing 1% BSA for 30 min at 20 °C–22 °C, and then stained with anti-mouse CD138 antibody (BioLegend). After incubation overnight at 4 °C, the slices were washed in PBS 5 times and incubated with Histofine Simple Stain MAX-PO (Nichirei Bio, Tokyo, Japan) for 30 min at 20 °C–22 °C. Peroxidase activity was detected with diaminobenzidine (Nichirei Bio). The slices were counterstained with hematoxylin and dehydrated. A Fluoview FV10i confocal laser scanning microscope (Olympus, Tokyo, Japan) or Biozero BZ-8100 (Keyence, Osaka, Japan) was used to acquire images.

### RNA extraction and quantitative real-time reverse-transcription polymerase chain reaction (qRT-PCR)

Cervical lymph nodes (cLNs) and SGs were submerged in approximately 5 volumes of RNAlater solution (Thermo Fisher Scientific) immediately after sampling. Spleens were strained through a 40-μm filter and subjected to red blood cell (RBC) lysis with 0.16 M of NH_4_Cl solution for 5 min. These samples or cultured NIH/3 T3 mouse fibroblast cells were suspended in Isogen (Nippon Gene, Tokyo, Japan). Total RNA was extracted using the Isogen method. Complementary DNA (cDNA) was synthesized at 37 °C for 15 min using a Prime Script reverse transcriptase master mix (Takara Bio, Shiga, Japan), and the reaction mixture was used for the PCR. qRT-PCR reactions were performed using a Taqman gene expression assay and normalized to glyceraldehyde-3-phosphate dehydrogenase *(Gapdh)* abundance. The primers and probes, all from Thermo Fisher Scientific, were as follows: *Gapdh*, Mm99999915_g1; *Ccr8*, Mm99999115-s1; *Ccl8*, Mm01297183-m1; *IL4*, Mm00445259_m1; *IL10*, Mm00439614_m1; and *Col1a2*, Mm00483888_m1.

### Enzyme-linked immunosorbent assay (ELISA)

Serum levels of mouse CCL8 were measured using a commercially available ELISA (R&D systems) according to the manufacturer’s instructions.

### Cell preparation for FACS staining

To obtain tissue-infiltrating mononuclear cells in the SGs, the SGs from LAT mice were harvested, cut into small pieces with scissors, and incubated at 37 °C for 30 min with 2 mg/mL collagenase D (Roche Diagnostics GmbH, Mannheim, Germany) and 5 mM CaCl_2_. After incubation, they were disrupted by grinding between frosted glass coverslips. After straining through a 40-μm filter and washing with the PBS, infiltrating mononuclear cells were isolated from parenchyma cells by OptiPrep (Cosmo Bio, Tokyo, Japan) with gradient centrifugation according to the manufacturer’s instructions.

### Fluorescence-activated cell sorting (FACS) staining

For FACS analyses, the cells were washed in FACS buffer (2% FBS in PBS) and then incubated with FACS antibodies. Staining for mouse CD4 and CD25 (all from BioLegend) was performed for 20 min using a mixture of antibodies. Dead cells were excluded by Fixable Viability Dye eFluor 780 (eBioscience, San Diego, CA). Intracellular staining for Foxp3, IFN-γ, IL-17, and IL-4 (all from BioLegend) was performed after fixation and permeabilization using a Foxp3/Transcription Factor Staining Buffer Set (eBioscience) according to the manufacturer’s instructions. Intracellular staining of IFN-γ, IL-17, and IL-4 (BioLegend) was performed after 4 h of stimulation by phorbol myristate acetate (PMA) (50 ng/mL), ionomycin (0.5 μg/ml), and GolgiStop (eBioscience) were added during the stimulation.

For phosphoextracellular signal-regulated kinase (ERK)1/2 (BioLegend) staining, Phosflow Lyse/Perm buffer and Perm Buffer III (Becton, Dickinson and Company [BD], Franklin Lakes, NJ) were used according to the manufacturer’s instructions. The samples were analyzed with a FACSVerse flow cytometer (BD), and the data were analyzed using FlowJo software (Tree Star, Ashland, OR). The relative mean fluorescence intensity (MFI) ratio was calculated on the basis of the ratio of the MFI for a marker to the MFI of its isotype control.

### In vivo inhibition of CCL8

One hundred micrograms of anti-mouse CCL8 neutralizing antibody (clone 17D6, generated by the Tokyo University of Pharmacy and Life Sciences, Tokyo) or isotype control (BioLegend) was intravenously injected on days 1 and 2 in 5-week-old LAT mice on the basis of a previous study [16]. On day 8 (6 weeks of age), the effects of treatment on the development of tissue lesions (spleen, cLNs, and SGs) were assessed.

### Statistical analysis

All the data were expressed as means ± SEMs. Differences between groups were examined for significance using a 2-tailed unpaired *t* test or Kruskal-Wallis test. Probability values lower than 0.05 were considered to indicate significant differences. Statistical analysis was performed using GraphPad Prism version 9 (GraphPad software, San Diego, CA).

## Results

### Histopathologic analysis of the salivary glands of 6-week-old LAT mice and littermates

**Fig. 1:**
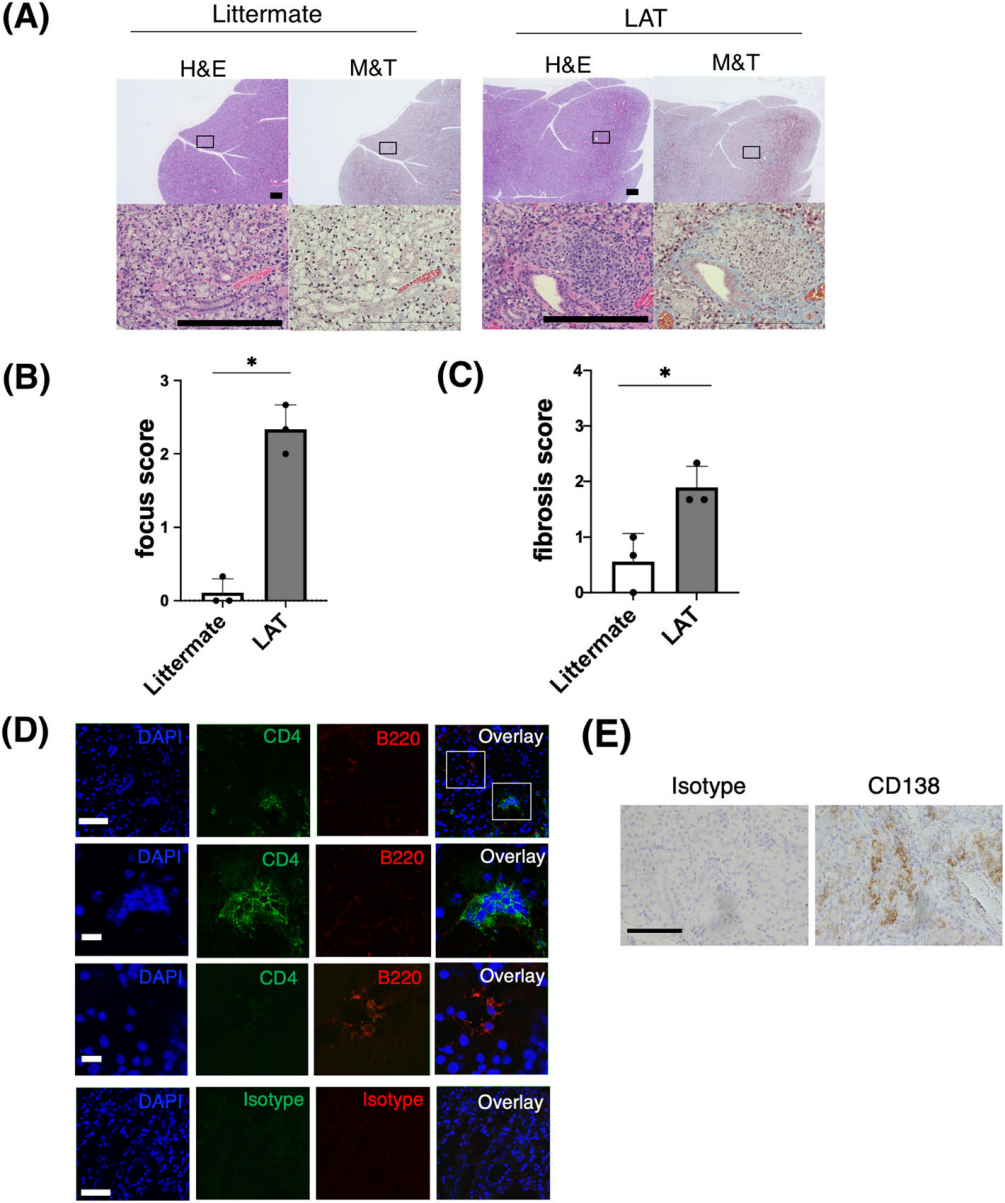
Histologic analysis of the salivary glands of 6-week-old LAT mice and of the littermates. **A** H&E staining and M&T staining of salivary glands. The boxed areas in the upper panels are shown in detail in the lower panels. Scale bars, 200 μm. Representative images of 3 mice per group. H&E, hematoxylin and eosin stain; M&T, Masson’s trichrome stain. **B** Focus score of salivary glands. Histologic evaluation was performed in a blinded manner (littermate, *n* = 3; LAT mice, *n* = 3). **C** Fibrosis score of salivary glands. Histologic evaluation was performed in a blinded manner (littermate, *n* = 3; LAT mice, *n* = 3). **D** Images are representative of salivary gland slices of LAT mice stained for CD4 (green), B220 (red), and 4′6-diamino-2-phenylindole (DAPI) (blue). Top: low magnification images; second row from the top: the part focused on CD4^+^ cells; third row from the top: the part focused on B220^+^ cells; bottom row: images stained for isotype control. Scale bars (top, 50 μm; second and third rows from the top, 10 μm; bottom row, 50 μm). **E** Salivary gland slices of LAT mice stained for CD138 or isotype control. Scale bar,100 μm. *p < 0.05, unpaired *t* test

We histopathologically analyzed the SGs of 6-week -old LAT mice and the control littermates. All the analyzed LAT mice displayed inflammatory lesions showing infiltration of mononuclear cells with fibrosis (Fig. 1A). The histologic focus scores and fibrosis scores of the LAT mice were significantly higher than those of the littermates (Fig. 1B, C). Immunostaining revealed infiltration of CD4^+^ T cells, B220^+^ B cells, and CD138^+^ plasma cells into the SGs of the LAT mice (Fig. 1D, E). Angiogenesis of the SGs was less noticeable in LAT mice. This is consistent with description in the 2019 American College of Rheumatology/European League Against Rheumatism classification criteria for IgG4-related disease in which angiogenesis is not adopted in the histopathology section [17].

### Upregulation of the CCL8–CCR8 axis in the salivary glands of LAT mice

**Fig. 2:**
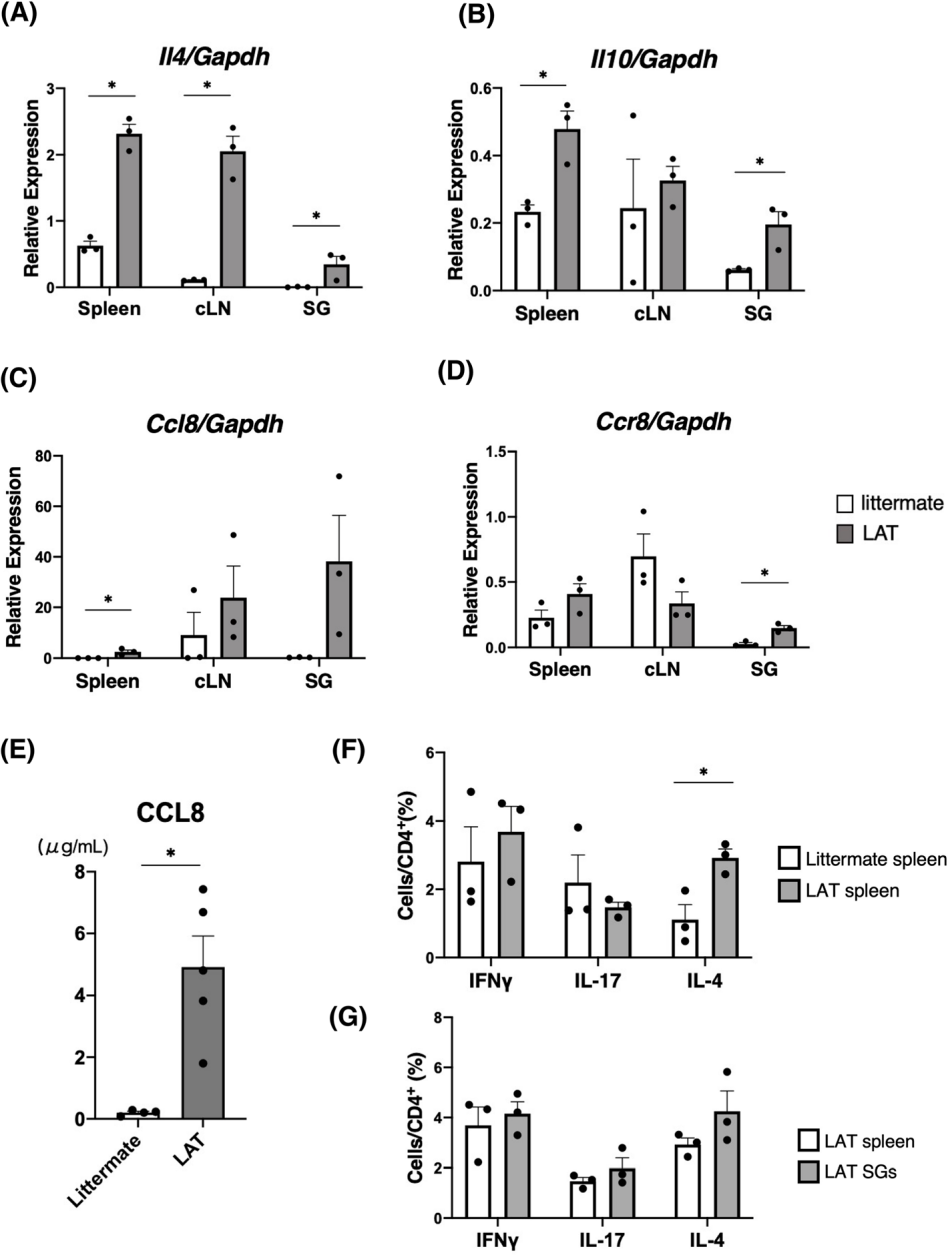
Upregulation of Th2 cytokines and the CCL8–CCR8 axis in 6-week-old LAT mice. **A** *Il4* mRNA levels in spleens, cervical lymph nodes (cLNs), and salivary glands (SGs) were determined by qRT-PCR. The expression levels were calculated as relative amounts normalized to *Gapdh* (littermate, *n* = 3; LAT, *n* = 3). **B** *Il10* mRNA levels in spleens, cLNs, and SGs were determined by qRT-PCR. The expression levels were calculated as relative amounts normalized to *Gapdh* (littermate, *n* = 3; LAT, *n* = 3). **C** *Ccl8* mRNA levels in each tissue were determined by means of qRT-PCR. The expression levels were calculated as relative amounts normalized to *Gapdh* (littermate, *n* = 3; LAT, *n* = 3). **D** *Ccr8* mRNA levels in each tissue was determined by means of qRT-PCR. The expression levels were calculated as relative amounts normalized to *Gapdh* (littermate, *n* = 3; LAT, *n* = 3). **E** Serum CCL8 levels were determined by ELISA (littermate, *n* = 4; LAT, *n* = 5). **F** Splenocytes from littermates and LAT mice were assessed by flow cytometry for cytokine production after stimulation with phorbol myristate acetate and ionomycin for 4 h. The proportion of CD4^+^ cells that producing each cytokine was quantified (littermate, *n* = 3; LAT, *n* = 3). **G** Splenocytes and mononuclear cells infiltrated in SGs from LAT mice were assessed by flow cytometry for cytokine production after stimulation with phorbol myristate acetate and ionomycin for 4 h. The proportion of CD4^+^ cells that producing each cytokine was quantified. *p < 0.05, Unpaired *t* test. The data are presented as means ± SEMs

To clarify whether Th2 cytokines and the CCL8–CCR8 axis are upregulated in LAT mice as they are in IgG4-RD patients, we compared *Il4*, *Il10, Ccl8*, and *Ccr8* mRNA expressions in the spleen, cLNs, and SGs of the LAT mice and the littermates. Our results showed that *Il4* expression in the spleen, cLNs, and SGs of the LAT mice was significantly higher than that in the littermates (Fig. 2A). *Il10* and *Ccl8* expressions were significantly higher in the spleen (Fig. 2B, C), and *Il10* and *Ccr8* expressions were significantly higher in the SGs of the LAT mice (Fig. 2D). Besides, serum CCL8 levels were significantly higher in the LAT mice than in the littermates (Fig. 2E). The proportion of IL-4^+^ cells in splenic CD4^+^ cells was significantly higher in LAT mice compared to littermates, whereas comparable in IFN-γ^+^ and IL-17^+^ cells (Fig. 2F and Figure S1[A][B]). There was no significant difference in the production of these cytokines between the spleen and SGs of LAT mice (Fig. 2G), while, in LAT mice, CD4^+^CD25^+^Foxp3^+^ cells significantly decreased compared with littermates (Figure S2[A][B]), which was consistent with the previous report [18].

### CCL8-expressing cells in the salivary glands of LAT mice

**Fig. 3:**
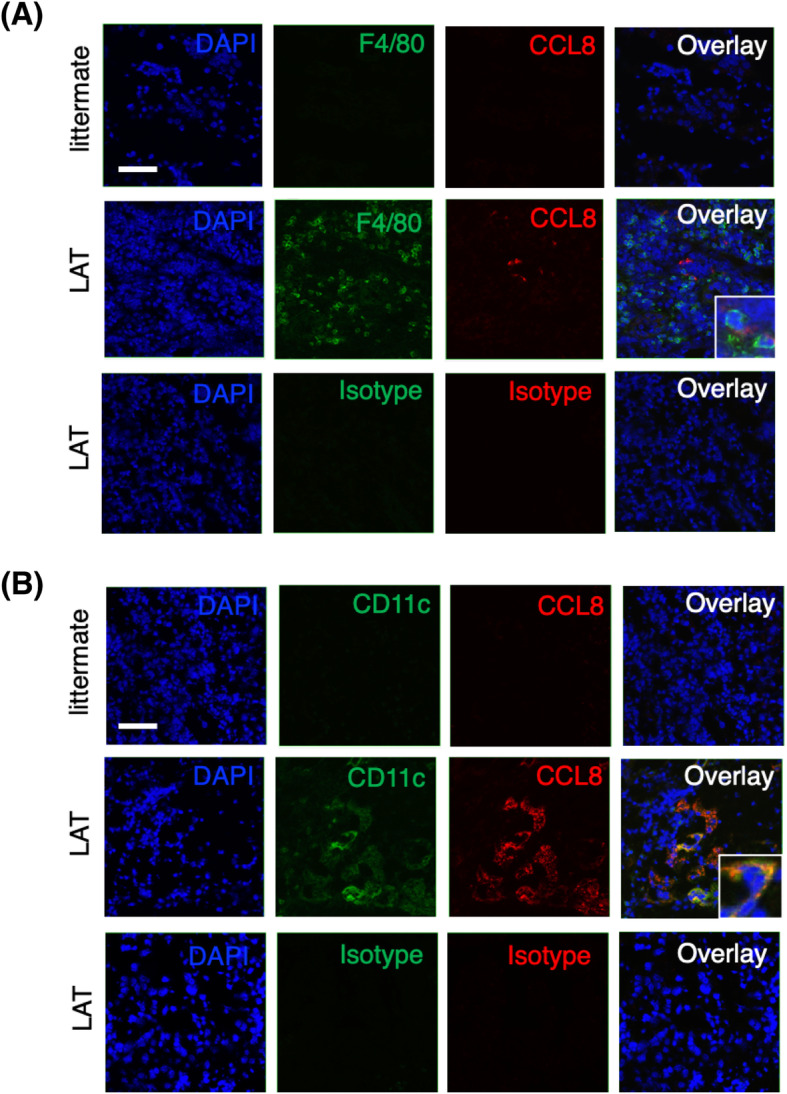
CCL8-expressing cells in salivary glands of LAT mice. **A** Salivary gland slices from 6-week-old control littermates or LAT mice were stained for F4/80 (green) and CCL8 (red). **B** Salivary gland slices from 6-week-old control littermates or LAT mice were stained for CD11c (green) and CCL8 (red). DAPI (blue), 4′6-diamino-2-phenylindole. Scale bars, 50 μm

To confirm that mCCL8-expressing cells were exactly present in the SGs, we immunofluorescently stained the infiltrating cells. The results revealed that CCL8-expressing F4/80-positive macrophages (Fig. 3A) and CD11c-positive dendritic cells (Fig. 3B) were predominantly present in the SGs of the LAT mice. In contrast, these cells were not detected in the littermates.

### Suppression of salivary gland inflammation and fibrosis in LAT mice by administration of anti-mCCL8 neutralizing antibody

**Fig. 4:**
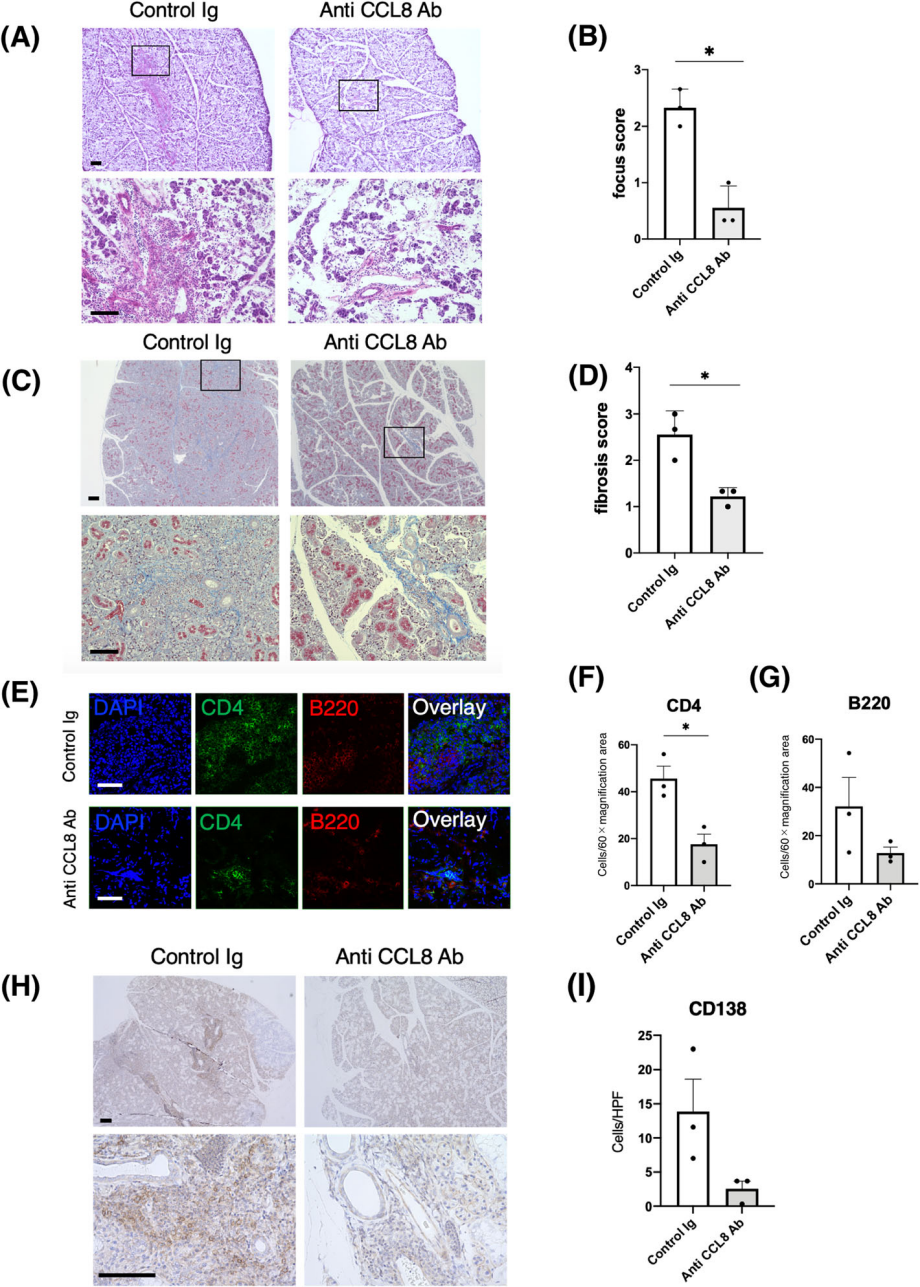
Suppression of salivary gland inflammation and fibrosis in LAT mice by CCL8 neutralizing therapy. **A** H&E staining of salivary glands in LAT mice treated with anti-CCL8 neutralizing antibody (anti-CCL8 Ab) or control immunoglobulin (control Ig). The boxed areas in the upper panels are shown magnified in the lower panels (scale bars: upper; 200 μm; lower, 100 μm). Representative images of 3 mice. **B** Comparison of focus score in salivary glands between anti-CCL8 Ab-treated LAT mice and control Ig-treated LAT mice. **C** M&T staining of salivary glands in LAT mice treated with anti-CCL8 Ab or control Ig. The boxed areas in the upper panels are magnified in the lower panels (scale bars: upper, 200 μm; lower, 100 μm). Representative images of 3 mice. **D** Comparison of fibrosis score in salivary glands of anti-CCL8 Ab-treated LAT mice and control Ig-treated LAT mice. **E** The salivary glands slices of LAT mice treated with anti-CCL8 Ab or control Ig were stained for CD4 (green) and B220 (red). DAPI (blue), 4′6-diamino-2-phenylindole (scale bars, 50 μm). **F** The average of CD4-positive cells per 60× magnification area. Three areas per mouse were counted. **G** The average of B220-positive cells per × 60 magnification area. Three areas per mouse were counted. **H** Salivary glands slices of LAT mice treated with anti-CCL8 Ab or control Ig were stained for CD138. Counterstaining was done with hematoxylin (scale bars: upper, 200 μm; lower, 100 μm). **I** The average of CD138-positive cells per high power field. Three areas per mouse were counted. The data are presented as the means ± SEMs of 3 mice. *p < 0.05, unpaired *t* test

To determine whether the mCCL8–CCR8 pathway could be a therapeutic target for IgG4-RD, we evaluated the SG pathology of anti-mCCL8 neutralizing antibody- (anti-CCL8 Ab) and control immunoglobulin- (control Ig) treated LAT mice. The development of sialadenitis was found to be inhibited in the anti-CCL8 Ab-treated LAT mice when compared with the control Ig-treated mice (Fig. 4A). The focus scores of the anti-CCL8 Ab-treated mice were significantly lower than those of the control Ig-treated mice (Fig. 4B). Interestingly, the development of SG fibrosis was also found to be inhibited in the anti-CCL8 Ab-treated LAT mice (Fig. 4C). The fibrosis scores of the anti-CCL8 Ab-treated mice were significantly lower than those of the control Ig-treated mice (Fig. 4D). Immunostaining revealed that the number of CD4^+^ cells that had infiltrated the region around the SG ducts of the anti-CCL8 Ab-treated mice was significantly reduced when compared with that of the control Ig-treated mice (Fig. 4E, F). The number of B220^+^ cells and CD138^+^ cells also tended to be reduced, although the difference between the treatment groups did not reach statistical significance (Fig. 4E, G, H, I).

### Effects of CCL8-blocking therapy on mRNA expression of Il4 and Il10 in LAT mice

**Fig. 5:**
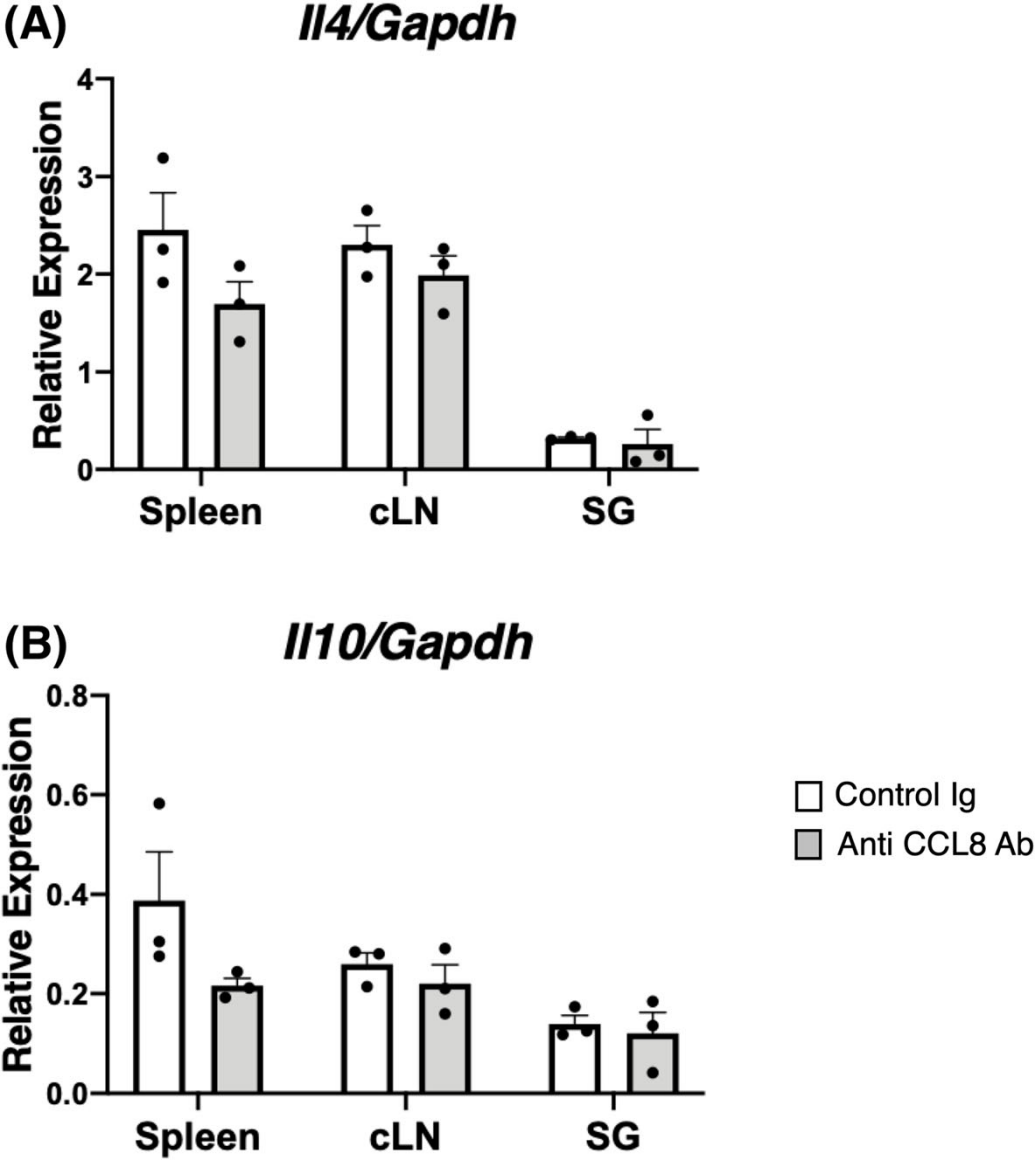
Effects of CCL8-blocking therapy on mRNA expression of *Il4* and *Il10* in LAT mice. **A** Comparison of mRNA expression levels of *Il4* in anti-CCL8 Ab-treated LAT mice and control Ig-treated LAT mice. **B** Comparison of mRNA expression levels of *Il10* in anti-CCL8 Ab-treated LAT mice and control Ig-treated LAT mice. cLN, cervical lymph nodes; SG, salivary glands. The data are presented as means ± SEMs of 3 mice. *p < 0.05, unpaired *t* test

Next, we investigated whether anti-mCCL8 neutralizing therapy affected the cytokine environment in LAT mice. The mRNA expression of *Il4* and *Il10* tended to be reduced in the anti-CCL8 Ab-treated LAT mice when compared with the control Ig-treated group, especially in the spleen, although the difference between the treatment groups did not reach statistical significance (Fig. 5A, B). These results could suggest that the attenuated Th2 phenotypes were not the main reason for the improved fibrosis. The following in vitro experiment was conducted to investigate the possibility that mCCL8 acts directly on fibroblasts and has a mechanism involved in fibrosis.

### Effects of mCCL8 on collagen production of fibroblasts in vitro

**Fig. 6:**
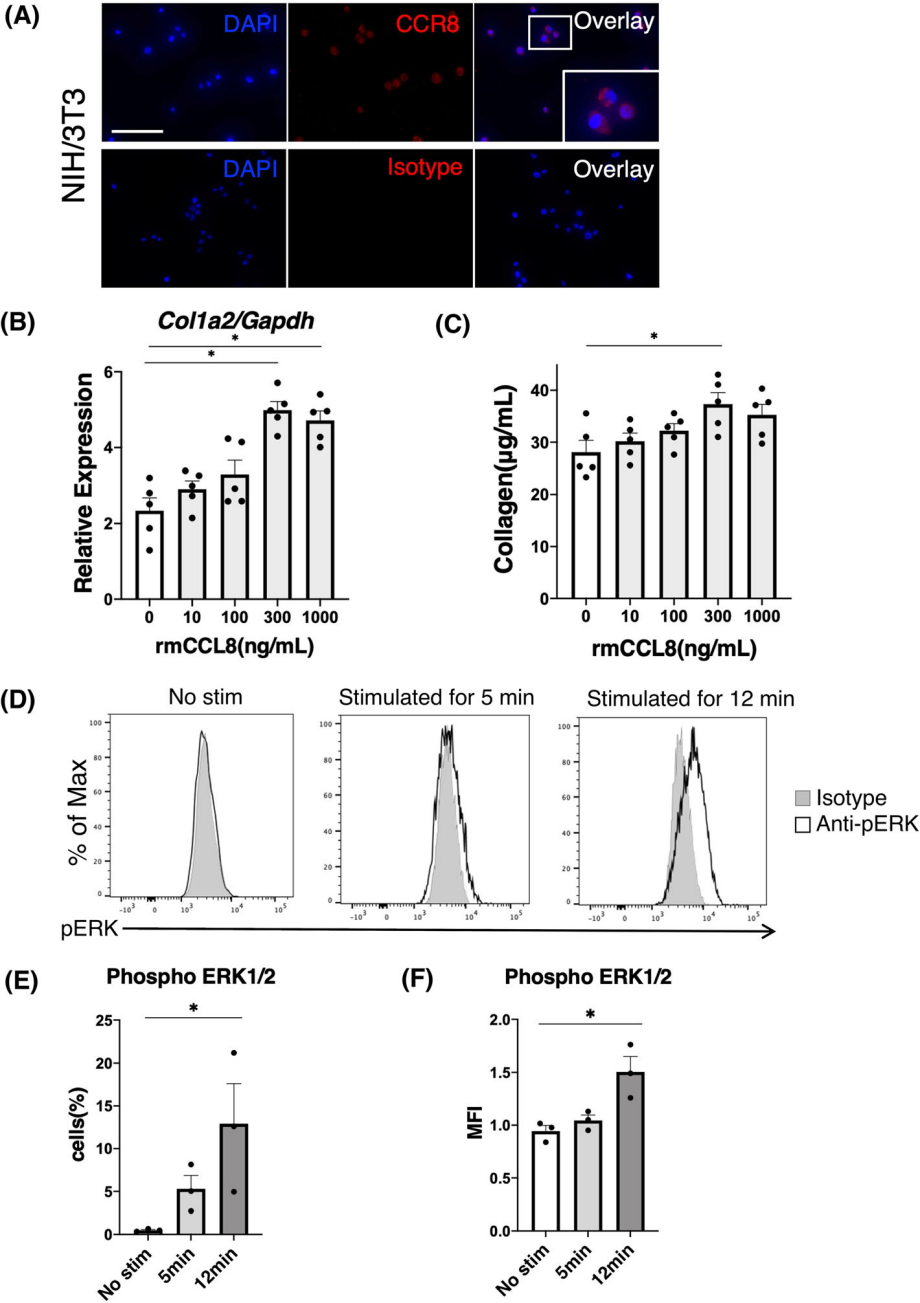
Effects of CCL8 on collagen production of fibroblasts in vitro. **A** Immunofluorescence staining of CCR8 in a mouse fibroblast cell line (NIH/3 T3). Upper panels: Alexa Fluor 546-conjugated anti-mouse CCR8 antibody staining. Lower panels: isotype control antibody staining. Original magnification × 40 (scale bar, 50 μm). DAPI, 4′6-diamino-2-phenylindole. **B** mRNA expression levels of *Col1a2* in fibroblasts treated with recombinant mouse CCL8 for 24 h. **C** Total collagen protein production from fibroblasts treated with recombinant mouse CCL8 for 24 h. **D** Flow cytometric analysis of phosphorylated ERK1/2 (pERK) in fibroblasts treated with recombinant mouse CCL8 at 300 ng/mL of concentration for the indicated times (min). The panels show a representative histogram of each of the 3 wells for the indicated times. **E** The bar graph shows the rate of pERK1/2-positive cells. **F** The bar graph shows the mean fluorescence intensity (MFI) of pERK1/2 in fibroblasts treated with recombinant mouse CCL8. The data are presented as means ± SEMs of 5 (**B** and **C**) and 3 (**E** and **F**) wells in each condition. *p < 0.05, Kruskal-Wallis test

To further analyze the association between mCCL8 and fibrosis, we cultured a mouse fibroblast cell line (NIH/3 T3) with or without recombinant mouse CCL8 (rmCCL8). First, we confirmed the protein expression of CCR8 in NIH/3 T3 cells by means of immunofluorescence staining (Fig. 6A). Then, the NIH/3 T3 cells were stimulated with various concentrations of rmCCL8. Twenty-four hours after the stimulation with rmCCL8, we investigated the mRNA expression level of *Col1a2* and the total collagen protein production in the NIH/3 T3 cells. The mRNA expression levels of *Col1a2* and the total collagen protein production were increased in rmCCL8 dose-dependent manner (Fig. 6B, C). To investigate whether ERK1/2 phosphorylation is involved in upregulation of collagen production by rmCCL8, the NIH/3 T3 cells were treated with 300 ng/mL of rmCCL8. After 12 min of stimulation with rmCCL8, phosphorylation of ERK1/2 in the NIH/3 T3 cells was significantly increased in both the MFI and the positive cell ratio when compared with no stimulation (Fig. 6D, E, F). Thus, our data suggested that the mCCL8–CCR8 pathway is involved in the induction of collagen production by fibroblasts via phosphorylation of ERK1/2.

## Discussion

In this study, we showed that LAT mice had plasmacyte infiltration and fibrosis of the SGs similar to human IgG4-RD as well as an enhanced CCL8–CCR8 axis. Neutralization of mCCL8 showed improvement of sialadenitis including fibrosis, and it was shown that mCCL8 may enhance collagen production in fibroblasts via phosphorylation of ERK1/2. Through our investigation, the following 3 clinicopathologic findings emerged.

First, commonalities between LAT mice and IgG4-RD have been further demonstrated. We confirmed that LAT mice had a type 2 cytokine-dominant immune environment that reported in IgG4-RD including IgG4-ralated sialadenitis [3], while Treg cells were decreased in LAT mice. Thus, we could interpret that LAT mice did not reflect enhanced Treg response, but Th2 response of IgG4-RD as one of the mimicking mice models for IgG4-RD. The expression levels of the mCCL8–CCR8 axis were upregulated in LAT mice, in common with those of hCCL18–CCR8 in IgG4-RD. Therefore, we thought that the potential of the mCCL8 (hCCL18)–CCR8 pathway as a therapeutic target could be clarified by using LAT mice as a model of IgG4-RD. The chemokine–chemokine receptor system in mice differs from that in humans [7]. In mice, CCL1 and CCL8 have been reported as ligands for CCR8 [14]. The relationship between hCCL18 and mCCL8, not mCCL1, has been proven to be a functional analog [19]. Focusing on the induction mechanism, IL-4 and IL-10 induce mCCL8 production from macrophages, and the same can be said for hCCL18 [14]. In LAT mice, infiltration of mCCL8-producing macrophages was observed in the SGs. Furthermore, both *Il4* and *Il10* were highly expressed in the SGs of LAT mice, and this environment may induce mCCL8 production.

Second, our results suggest that the CCL8–CCR8 axis may have a potential as a therapeutic target for IgG4-RD. The improvement of inflammatory cell infiltration was thought to be due to the suppression of chemotaxis of T cells and B cells to the SGs in which mCCL8 was produced by macrophages and dendritic cells. Expression of CCR8 in T cells was reportedly upregulated in the Th2 cytokine environment [14]. Since LAT mice showed a Th2 cytokine-dominant phenotype, like that of IgG4-RD [11], it is an environment in which CCR8 expression in T cells is likely to be enhanced. Even more interesting, anti-CCL8 Ab improved not only the infiltration of inflammatory cells but also the fibrosis of the SGs. Thus, the CCL8–CCR8 axis may have pathogenic roles beyond the chemotaxis of lymphocytes, such as induction of fibrosis.

Third, we showed for the first time that mCCL8 could induce collagen production in fibroblasts via ERK1/2 phosphorylation. Although many unclear points about the downstream pathway of CCR8 remained, it was reported that ERK phosphorylation occurred when a signal from the ligand was input in this pathway [20, 21]. ERK was contained in mitogen-activated protein kinase (MAPK) [22], which transmitted extracellular stimuli into cells, and ERK1/2 has been reported to induce fibroblast proliferation [23]. Still, only a limited amount of evidence is available related to the function of hCCL18 in inducing collagen production in fibroblasts [8], and it was reported that the Th2 cytokine environment itself was involved in the induction of fibrosis [24]. Importantly, our data suggest that mCCL8 could induce collagen production in fibroblasts via an ERK-dependent pathway, independently of Th2 cytokines.

The following has been reported regarding the fibrotic mechanism in human IgG4-RD. CD4^+^ T cells with cytotoxic activity (CD4^+^CTL), which were clonally proliferated in the affected organs, expressed cytotoxic proteins such as granzyme B and perforin [25]. These cells produce IL-1β, IFNγ, and TGFβ, which activates fibroblasts and alternatively activated (M2) macrophages, thereby inducing fibrosis in IgG4-related dacryoadenitis and sialadenitis [26]. Moreover, the number of infiltrating M2 macrophages has been reported to correlate with the fibrosis score in IgG4-related sialadenitis and that these macrophages produce hCCL18 [27]. These reports also supposed that hCCL18 acted as a profibrotic factor and that the hCCL18–CCR8 axis may be a therapeutic target for IgG4-RD.

The role of chemokines in directing T cell polarization in addition to their role in chemoattraction has been shown in several reports; for example, CXCL10 for T helper type-1 cells [28, 29], CXCL11 and CXCL12 for T regulatory-1 cells [30, 31], and CCL1 for FOXp3^+^ Treg cells [20] have been reported to be involved in the polarization and differentiation of CD4^+^ T cells. Analysis of the effect of the mCCL8 (hCCL18)–CCR8 axis on CD4-positive T cell differentiation needs to be further investigated. Moreover, we hope that our research findings to lead to the development of treatments targeting the hCCL18–CCR8 pathway as a rescue therapy for intractable IgG4-RD cases such as steroid-resistant cases and recurrent cases.

## Conclusions

In conclusion, we have demonstrated that upregulation of the mCCL8 (analog of hCCL18)–CCR8 axis in LAT mice could play a crucial role in fibrosis via ERK1/2 pathway-dependent type-I collagen production, as well as in cell infiltration in sialadenitis. In fact, anti-CCL8 Ab improved both the inflammation and the fibrosis of SGs in LAT mice, suggesting that inhibition of the hCCL18 (mCCL8)–CCR8 axis should shed light on new therapies for IgG4-RD.

## Supplementary Information

**Additional file 1: Figure S1.** Flow cytometric analysis of spleen and SGs for cytokine production. (A) Gating strategy for flow cytometric analysis of CD4^+^ T cells. Images are representative results of splenocyte from littermate mice. (B) FACS plot for production of IFN-γ, IL-17, and IL-4 by CD4^+^ cells derived from 6-week-old littermates and LAT mice after stimulation with phorbol myristate acetate and ionomycin for 4 hours. CD4^+^ gated cells were shown in FACS plot. FACS plot is a representative of analysis of six mice (littermate, n=3; LAT, n=3).

**Additional file 2: Figure S2.** CD25 and Foxp3 expression in splenocytes from littermate and LAT mice. (A) FACS analysis of CD4, CD25, and Foxp3 in splenocyte from 6-week-old littermates and LAT mice. Dead cells were gated out by using fixable viability dye. FACS plot is a representative of analysis of six mice (littermate, n=3; LAT, n=3). (B) The proportion of CD4^+^CD25^+^Foxp3^+^ cells were compared between littermates and LAT mice. *p<0.05, Unpaired *t* test. The data are presented as means ± SEMs.

## Data Availability

The data that support the findings of this study are available from the corresponding author upon reasonable request.

## Abbreviations

IgG4-RD: Immunoglobulin G4-related disease
LAT: The linker for activation of T cells
LAT mice: LAT Y136F knockin mice
SGs: Salivary glands
anti-CCL8 Ab: Anti-CCL8 neutralizing antibody
rmCCL8: Recombinant mouse CCL8
LSGs: Labial salivary glands
mCCL8: Murine CCL8
hCCL18: Human CCL18
Th2: Type-2 T helper
DMEM: Dulbecco’s modified Eagle’s medium
FBS: Fetal bovine serum
H&E: Hematoxylin and eosin
M&T: Masson’s trichrome
PBS: Phosphate-buffered saline
qRT-PCR: Quantitative real-time reverse-transcription polymerase chain reaction
cLNs: Cervical lymph nodes
RBC: Red blood cell
cDNA: Complementary DNA
Gapdh: Glyceraldehyde-3-phosphate dehydrogenase
ELISA: Enzyme-linked immunosorbent assay
FACS: Fluorescence-activated cell sorting
PMA: Phorbol myristate acetate
ERK: Extracellular signal-regulated kinase
MFI: Mean fluorescent intensity
control Ig: Control immunoglobulin
MAPK: Mitogen-activated protein kinase
CD4^+^CTL: CD4^+^ T cells with cytotoxic activity
