# Supplementary figures and images for: Pathogenic roles and therapeutic potential of the CCL8–CCR8 axis in a murine model of IgG4-related sialadenitis
Source: Arthritis Res Ther. 2021 Aug 14;23:214. doi: 10.1186/s13075-021-02597-6 (PMC8364087; doi:10.1186/s13075-021-02597-6)

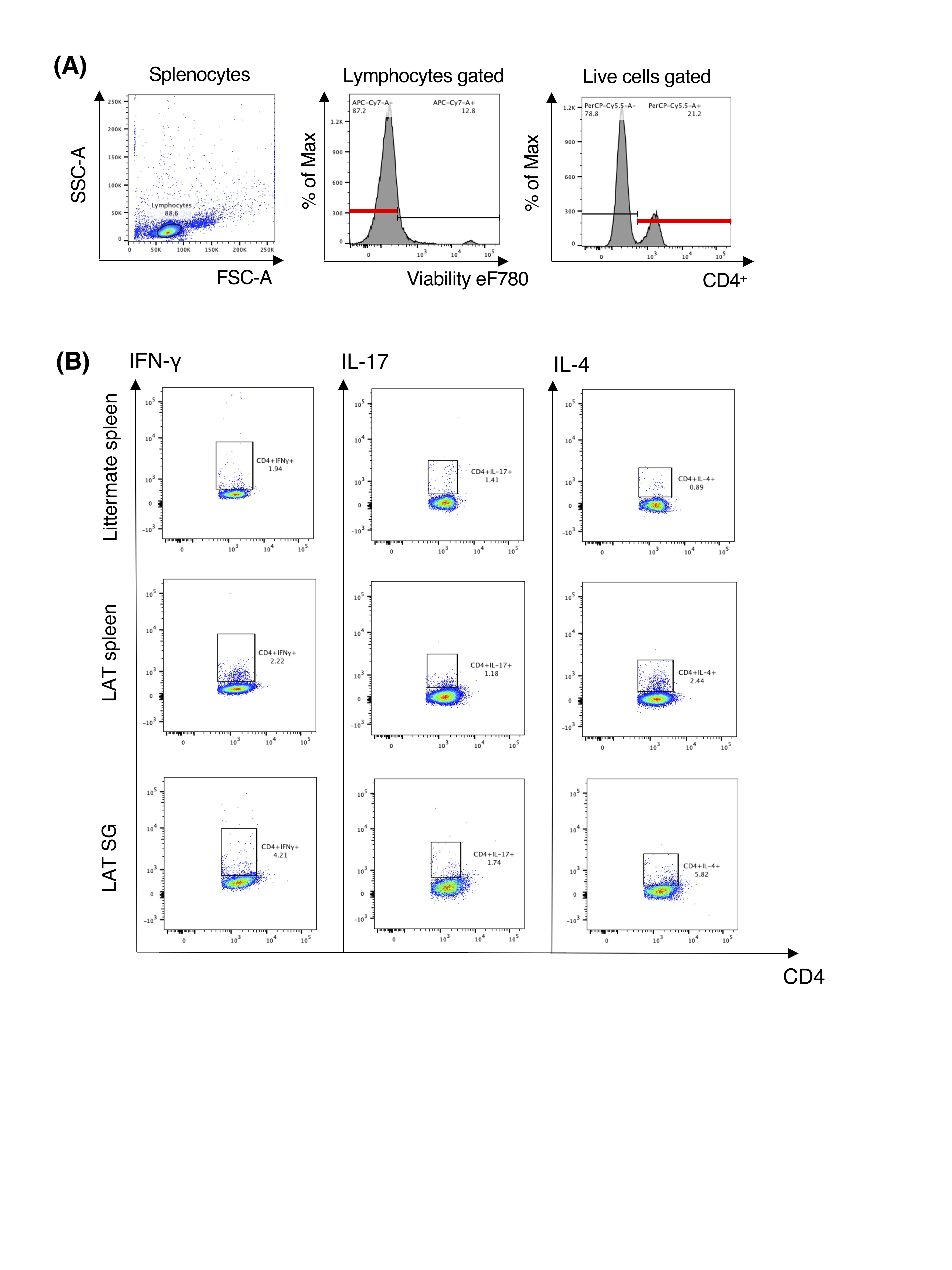

Supplement: Supplementary file 1 — Additional file 1: Figure S1. Flow cytometric analysis of spleen and SGs for cytokine production. (A) Gating strategy for flow cytometric analysis of CD4+ T cells. Images are representative results of splenocyte from littermate mice. (B) FACS plot for production of IFN-γ, IL-17, and IL-4 by CD4+ cells derived from 6-week-old littermates and LAT mice after stimulation with phorbol myristate acetate and ionomycin for 4 hours. CD4+ gated cells were shown in FACS plot. FACS plot is a representative of analysis of six mice (littermate, n=3; LAT, n=3). [file 13075_2021_2597_MOESM1_ESM.tiff]

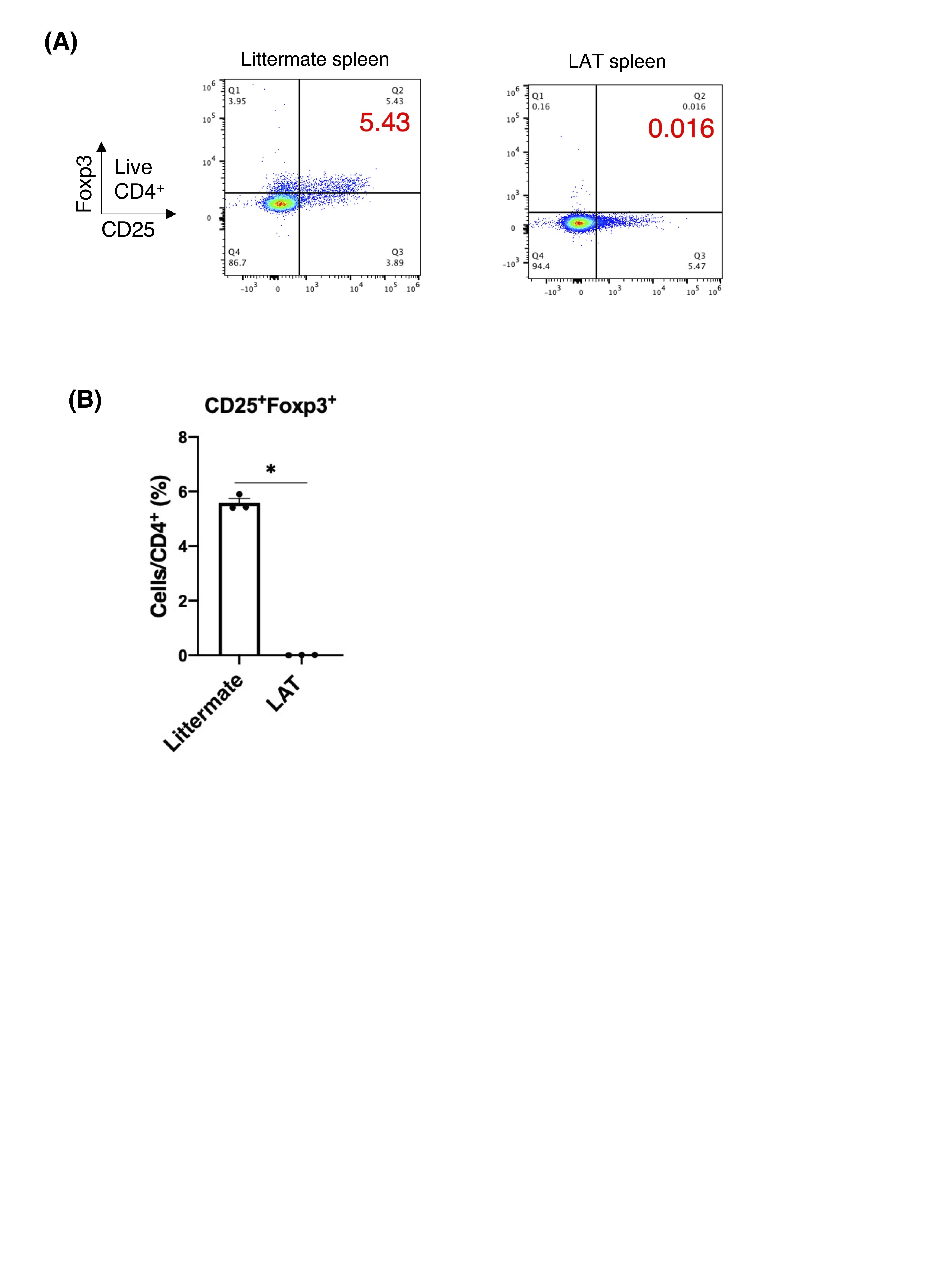

Supplement: Supplementary file 2 — Additional file 2: Figure S2. CD25 and Foxp3 expression in splenocytes from littermate and LAT mice. (A) FACS analysis of CD4, CD25, and Foxp3 in splenocyte from 6-week-old littermates and LAT mice. Dead cells were gated out by using fixable viability dye. FACS plot is a representative of analysis of six mice (littermate, n=3; LAT, n=3). (B) The proportion of CD4+CD25+Foxp3+ cells were compared between littermates and LAT mice. *p<0.05, Unpaired t test. The data are presented as means ± SEMs. [file 13075_2021_2597_MOESM2_ESM.tiff]
